# Spatial inference of ancestor locations suggests northern refugia for canopy‐forming kelps in the Pacific Northwest

**DOI:** 10.1111/nph.71293

**Published:** 2026-06-01

**Authors:** Jordan B. Bemmels, Kristy J. Kroeker, Stephen R. Palumbi, Rachael A. Bay, Kristen M. Gruenthal, Sandra C. Lindstrom, Matthew M. Osmond, Gregory L. Owens

**Affiliations:** ^1^ Department of Biology University of Victoria Victoria BC V8P 5C2 Canada; ^2^ The Kelp Rescue Initiative Bamfield Marine Sciences Centre Bamfield BC V0R 1B0 Canada; ^3^ Department of Ecology and Evolutionary Biology University of California Santa Cruz Santa Cruz CA 95060 USA; ^4^ Hopkins Marine Station, Department of Biology Stanford University Pacific Grove CA 93950 USA; ^5^ Department of Evolution and Ecology University of California Davis Davis CA 95616 USA; ^6^ Division of Commercial Fisheries Alaska Department of Fish and Game Juneau AK 99802 USA; ^7^ Department of Botany University of British Columbia Vancouver BC V6T 1Z4 Canada; ^8^ Department of Ecology and Evolutionary Biology University of Toronto Toronto ON M5S 3B2 Canada

**Keywords:** ancestral recombination graph, kelp forest, *Macrocystis*, *Nereocystis*, northern refugia, phylogeography, range expansion

## Abstract

- Pockets of the formerly glaciated Pacific coastline of North America likely remained ice‐free throughout the Last Glacial Maximum (LGM). These areas may have served as refugia for terrestrial species, but less is known about their role in the persistence of marine plants and other coastal species.
- We examined genetic diversity from > 1000 newly and previously sequenced whole genomes of canopy‐forming kelps of the genera *Nereocystis* and *Macrocystis* and built simple ecological niche models. We then reconstructed ancestral recombination graphs and modeled the geographic locations of genetic ancestors through time.
- We detected high genetic diversity in both species in north‐central British Columbia, in a region where suitable LGM habitat is plausible. Ancestor locations spatially converged backward in time toward this region, with multiple refugia inferred between northern Vancouver Island and southern Haida Gwaii. An expanded set of global samples for *Macrocystis* confirmed pre‐LGM divergence with California but hinted at the possibility of subsequent gene flow.
- *Nereocystis* and *Macrocystis* survived glaciation in northern refugia. The northern persistence of these foundation species raises the possibility that biodiverse kelp forest ecosystems could have continuously occupied portions of the northern Pacific coastline since the LGM.

## Graphical abstract

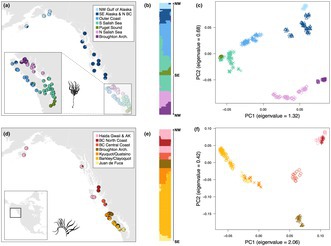

## Introduction

Range shifts associated with Pleistocene glacial cycles have profoundly impacted species distributions and geographic patterns of genetic diversity (Hewitt, 2000; Soltis *et al*., 2006). Characterizing range shifts has been critical to explaining the evolution of genetically distinct lineages (Pamilo & Savolainen, 2004), understanding community assembly (Cortés‐Guzmán *et al*., 2024), and prioritizing areas of elevated and endemic genetic diversity for conservation (Hampe & Petit, 2005; Médail & Diadema, 2009). Early studies of temperate terrestrial species emphasized a general pattern of range contraction during glacial periods into low‐latitude refugia and postglacial range expansion to higher latitudes as ice sheets receded (Davis, 1983; Hewitt, 2000). Founder effects during range expansion are expected to cause decreasing genetic diversity along the path of migration (Hewitt, 2000), but elevated diversity has also been observed in areas of secondary contact between genetically differentiated lineages originating from separate refugia (Petit *et al*., 2003).

In recent decades, traditional expectations of large southern refugia for temperate Northern Hemisphere terrestrial species have been challenged by the inferred existence of smaller northern refugia located in unique microclimates close to glacial margins and surrounded by otherwise inhospitable climate (Stewart & Lister, 2001; Provan & Bennett, 2008; Lumibao *et al*., 2017; Hošek *et al*., 2024). The debate about the relative size and latitude of refugia has been extended to coastal marine species (Maggs *et al*., 2008; Neiva *et al*., 2016), where support has been found for both low‐ (Assis *et al*., 2018) and high‐latitude refugia (Neiva *et al*., 2020; Bringloe *et al*., 2022; Jaugeon *et al*., 2025). Nonetheless, the refugial histories of many regions remain subject to debate. One such region (Mann & Gaglioti, 2024) is the formerly glaciated Pacific coastline of North America (Supporting Information Fig. S1). The Last Glacial Maximum (LGM) occurred asynchronously along different regions of this coastline, with local maxima reached from 24 to 15 ka (Mann & Hamilton, 1995; Mann & Gaglioti, 2024). During local maxima, terrestrial ice sheets largely extended up to or beyond the coastline (Mann & Hamilton, 1995), but several coastal pockets may have remained continuously unglaciated (Mann & Gaglioti, 2024). Geological and fossil evidence has suggested small unglaciated regions in the southern Alexander Archipelago (Carrara *et al*., 2007), Haida Gwaii (Warner *et al*., 1982; Mathewes & Clague, 2017), the central coast of British Columbia (BC; Shaw *et al*., 2020), and northern Vancouver Island (Hebda *et al*., 2022), though these interpretations are not without controversy (Walcott *et al*., 2022; Mann & Gaglioti, 2024).

Genetic evidence has sometimes supported the idea that these putatively unglaciated pockets along the Pacific coastline served as refugia for terrestrial (Shafer *et al*., 2010) and marine (Jacobs *et al*., 2004; Marko *et al*., 2010) species. Terrestrial refugia in the southern Alexander Archipelago and Haida Gwaii may have hosted diverse taxa (Shafer *et al*., 2010) including large and small mammals (Heaton *et al*., 1996; Byun *et al*., 1997; Sawyer *et al*., 2019; Colella *et al*., 2021), birds (Pruett *et al*., 2013; Geraldes *et al*., 2019), freshwater fish (McCusker *et al*., 2000), and various angiosperms and ferns (Soltis *et al*., 1997). Marine species that may have survived off adjacent coastlines include intertidal fish and invertebrates (Hickerson & Ross, 2001; Hickerson & Cunningham, 2005; Marko *et al*., 2010; Pagowski *et al*., 2025; Curcio *et al*., 2026), salmon (Smith *et al*., 2001; Xuereb *et al*., 2022), and red and brown algae (Lindstrom *et al*., 1997; Gierke *et al*., 2023). Additional marine refugia may have been located in the central coast of BC (Lindstrom *et al*., 2021, 2025) and the Gulf of Alaska (Grant *et al*., 2020; Grant & Bringloe, 2020; Grant & Chenoweth, 2021).

Relative to terrestrial taxa from the northern Pacific coastline of North America, marine species have been less frequently studied and early phylogeographic studies often revealed contradictory results (Marko *et al*., 2010). The application of modern genomic perspectives to marine taxa has continued to reveal taxon‐specific scenarios: a southern (Cascadian) refugium in pink salmon (Tarpey *et al*., 2018), possible but uncertain northern refugia in other fish and invertebrates (Candy *et al*., 2015; Xuereb *et al*., 2022; Pagowski *et al*., 2025; Curcio *et al*., 2026), and no signatures of refugial expansion in puffins (Graham *et al*., 2023). In marine plants specifically, recent phylogeographic studies have typically not adopted a genomic perspective (Grant *et al*., 2020; Grant & Bringloe, 2020; Grant & Chenoweth, 2021; Gierke *et al*., 2023), while genomic studies have not focused on phylogeographic questions (Bemmels *et al*., 2025; Bringloe *et al*., 2025). Whether northern refugia scenarios inferred for other taxa are also widely applicable to temperate marine plants remains an open question.

Genomic studies would be well‐suited to address outstanding controversies (Walcott *et al*., 2022; Mann & Gaglioti, 2024) about the potential importance of northern refugia to the phylogeographic history of marine plants and other taxa from the Pacific coast of North America. Ideally, genomic studies would leverage recent conceptual and statistical advances that allow characterization of past species distributions and migration histories in previously unprecedented detail. Such advances have included, for example, the development of the directionality index (ψ) to infer the direction of range expansion (Peter & Slatkin, 2013, 2015); the integration of demographic and genetic models to infer the origins of range expansions and other parameters in a spatial landscape (He *et al*., 2017; Becheler & Knowles, 2022); and the emerging use of ancestral recombination graphs (ARGs; Griffith & Marjoram, 1996; Wiuf & Hein, 1999) to reconstruct the recombination and coalescence history of all loci across the genome (reviewed in Lewanski *et al*., 2024; Nielsen *et al*., 2025) and then infer the geographic locations of genetic ancestors through time (Wohns *et al*., 2022; Osmond & Coop, 2024; Deraje *et al*., 2025; Grundler *et al*., 2025). Studies that focus on widespread, ecologically important taxa as representatives of coastal marine ecosystems would be particularly valuable.

Kelp forests represent one of the major temperate marine ecosystems of Pacific North America. The two canopy‐forming species from this region are bull kelp (*Nereocystis luetkeana*), distributed from central California to southwest Alaska, and giant kelp (*Macrocystis* spp.), distributed from Baja California to southern Alaska as well as in the Southern Hemisphere (Macaya & Zuccarello, 2010). Like other kelps, these foundation species create habitat for highly biodiverse communities (Teagle *et al*., 2017) and promote ecosystem function through nutrient cycling and high primary productivity (Wernberg *et al*., 2019; Eger *et al*., 2023). Both species have been culturally and economically important to humans for many millennia (Turner, 2001; Dillehay *et al*., 2008), perhaps – it has been speculated – even providing vital resources to support initial human migration into North America (Erlandson *et al*., 2007, 2015; Braje *et al*., 2017). Previous research on *Nereocystis* and *Macrocystis* has suggested divergent phylogeographic histories. A Haida Gwaii or Southeast Alaska refugium was inferred for *Nereocystis* based on high genetic diversity of seven microsatellite markers (Gierke *et al*., 2023), though diversity was fairly high along many regions of the outer coast. By contrast, Assis *et al*. (2023) considered all *Macrocystis* populations north of Oregon to be of postglacial origin based on low genetic diversity among six microsatellite loci and a global ecological niche model. However, few northern populations were sampled and genetic diversity was higher in Alaska than BC, hinting that the postglacial history of *Macrocystis* may be more complicated than range expansion from a single southern refugium.

Phylogeographic inference in *Macrocystis* is further complicated by the existence of four holdfast morphologies, whose genetic relationships are only partially understood (Gonzalez *et al*., 2023; Lindstrom, 2023; Gonzalez & Raimondi, 2024). Of the two morphs present in North America, the *pyrifera* morph occurs from Baja to central California and disjunctly in portions of Alaska (Gonzalez *et al*., 2023) and Haida Gwaii (Saunders & McDevit, 2014); the *integrifolia* morph occurs from central California to Alaska (Macaya & Zuccarello, 2010). Both morphs also occur in the Southern Hemisphere. Whether morphs represent ecotypes of a monotypic global species, *M. pyrifera* (Demes *et al*., 2009) or multiple species (Lindstrom, 2023) remains controversial. It was recently demonstrated that *integrifolia* and *pyrifera* morphs from the same locality in California are genetically distinguishable (Gonzalez *et al*., 2023) and that holdfast morphology is genetically determined (Gonzalez & Raimondi, 2024). Current taxonomy considers all *Macrocystis* north of Point Conception, California, to be *M. tenuifolia*, but recognizes that genetic relationships within the Northern Hemisphere remain unclear (Lindstrom, 2023). Genetic analysis of populations sampled throughout North America could help clarify whether disjunct northern *pyrifera* are closely related to Californian *pyrifera*, which would suggest the need to account for a complex phylogeographic history separate from northern *integrifolia*.

To reconstruct the history of canopy‐forming kelp forests in the formerly glaciated Pacific coastline of North America, we analyze newly and previously sequenced whole genomes of *Nereocystis* and *Macrocystis* from Alaska, BC, and Washington (which we hereafter collectively refer to as the ‘Pacific Northwest’, defined broadly) and globally for *Macrocystis*. We characterize patterns of genetic diversity, divergence, and gene flow among populations and construct ARGs to infer population demographic history and the locations of genetic ancestors. Our main aims are (1) to infer whether refugia were located only in southern areas or also in northern areas along glacial margins, (2) to identify specific refugial locations, and (3) to test whether Pacific Northwest and California *Macrocystis* populations of either morph have been in recent genetic contact by estimating divergence times and testing for gene flow.

## Materials and Methods

### DNA sampling and sequencing

We analyzed 505 *Nereocystis* Postels & Ruprecht and 522 *Macrocystis* (L.) C.Agardh whole‐genome sequences (Tables S1, S2) from previously published datasets and *de novo* sequencing. We newly sequenced 56 *Nereocystis* and 21 *Macrocystis* from Alaska using previously described protocols (Bemmels *et al*., 2025). Briefly, we extracted DNA from silica‐dried blade tissue using a custom CTAB method (Bemmels *et al*., 2025) and prepared libraries for *Illumina* sequencing using *Integrated DNA Technologies (IDT)* xGen DNA Library Prep EZ Kits. Paired‐end 150‐bp libraries were sequenced on an *Illumina* NovaSeq X at Canada's Michael Smith Genome Sciences Centre (Vancouver). In addition, we performed low‐depth whole‐genome sequencing (for further details see Methods S1) of 137 new *Macrocystis* samples from California (Table S1), with libraries prepared following Rowan *et al*. (2019).

The North American *Macrocystis* samples represented in this study are a mixture of verified *integrifolia*, verified *pyrifera*, and unknown morphs (Supporting Information, Tables S1, S2). In California, all previously sequenced populations we analyzed are *pyrifera* morphs, except for a single *integrifolia* population from Stillwater Cove (Gonzalez *et al*., 2023). Morph identity was not assessed for any of the newly sequenced California populations. Morph identity was also not assessed for any previously sequenced BC and Washington populations, but these individuals are presumably mostly *integrifolia* as this is the primary morph from this region, except in Haida Gwaii where both morphs are known (Saunders & McDevit, 2014). By contrast, all newly sequenced Alaska populations are *pyrifera* morphs (S.C. Lindstrom, personal communication).

### SNP genotyping

We generated single nucleotide polymorphism (SNP) genotypes following Bemmels *et al*. (2025). We removed adapter contamination and filtered raw reads using default parameters in fastp v.0.23.2 (Chen *et al*., 2018) and aligned reads to reference genomes for *Nereocystis* (NCBI: GCA_031213475.1; Alves‐Lima *et al*., 2025) and *Macrocystis* (JGI PhycoCosm: *Macrocystis pyrifera* CI_03 v1.0; Diesel *et al*., 2023) using default parameters in bwa‐mem 0.7.18‐r1243 (Li, 2013). We merged all aligned reads for each individual and removed duplicates using SAMtools v.1.22.1 (Danecek *et al*., 2021) and picard v.2.26.3 (Broad Institute, 2024), respectively. To correct for read misalignments around indels, we realigned reads using the *RealignerTargetCreator* and *IndelRealigner* tools from Gatk v.3.8 (Van der Auwera & O'Connor, 2020).

We called genotypes using BCFTools v.1.17‐1.22 (Danecek *et al*., 2021) with the commands *bcftools mpileup ‐Q 30 ‐q 30* and *bcftools call ‐m ‐v*, and filtered calls to biallelic SNPs with the command *bcftools view ‐v snps ‐m 2 ‐M 2 ‐q 0.000001:minor*. To remove sites that likely represent alignment errors and confounded loci, we (1) used *BCFTools* to remove sites with >50% heterozygosity; (2) retained only SNPs passing additional quality metrics (Segregation Based Metric, SGB ≥ 2; −1.96 ≤ *x* ≤ 1.96, where *x* is each of Mapping Quality Bias, Mapping Quality vs Strand Bias, and Read Position Bias, or MQBZ, MQSBZ, and RPBZ, respectively; and Variant Distance Bias, VDB ≥0.05), following Bemmels *et al*. (2025); and (3) removed sites above the 98^th^ percentile of overall depth.

To this set of quality‐filtered sites, we subsequently applied missing‐data filters. We created datasets retaining individuals with a mean depth ≥ 8× for both species. For *Macrocystis*, we additionally created a dataset with mean depth ≥ 1×, in order to include the low‐depth samples from California. We then set genotypes to missing for all sites below 8× or 1× coverage, depending on the dataset, using the *bcftools + setGT* plugin, and removed all sites with ≥ 20% missing data. We hereafter refer to these datasets as the min8× and min1× datasets, respectively. We used the min8× datasets for all analyses, unless otherwise specified. For all datasets, we retained only autosomal scaffolds and contigs ≥ 1.5 Mbp in length.

We then identified and removed genetically identical individuals and close relatives using ngsrelate v.2 (Hanghøj *et al*., 2019). We ran *ngsRelate* on individual VCF files for each population and examined the pairwise relatedness *r*_ab_ (Hedrick & Lacy, 2015; Hanghøj *et al*., 2019). We considered close relatives to be those where *r*_ab_ > 0.3536. Although our study species frequently inbreed (Bemmels *et al*., 2025), in the absence of inbreeding, *r*_ab_ approximates twice the coefficient of kinship (Hedrick & Lacy, 2015). Thus, the threshold 0.3536 distinguishes first‐degree from second‐degree relatives (Manichaikul *et al*., 2010) with expected *r*_ab_ = 0.5 and 0.25, respectively. For clusters of related individuals, we retained only the individual with the smallest proportion of missing genotypes. Our final standard dataset sizes were as follows: min8× *Nereocystis*: 470 individuals, 16 628 760 SNPs, median depth 19.1×; min8× *Macrocystis*: 311 individuals, 11 091 173 SNPs, median depth 14.6×; min1× *Macrocystis*: 484 individuals, 12 639 657 SNPs, median depth 12.0×. We also created datasets of invariant sites using the same methods as above, except that we removed the ‐v flag from *bcftools call*, retained only sites with one unique allele using *bcftools view ‐Q 0.000001:nonmajor ‐V indels*, and did not apply any filters that are applicable to polymorphic sites only.

Some downstream analyses required removing selfed individuals, assuming that selfing reflects dispersal limitations rather than non‐random mating in kelp (Gaylord *et al*., 2006; Edwards, 2022). We identified selfed individuals from runs of homozygosity (ROHs) using *bcftools roh*. We removed repetitive regions of the genome (previously identified by Bemmels *et al*., 2025) from input VCFs using the *intersect* command in BEDTools v.2.30.0 (Quinlan & Hall, 2010). We then calculated the inbreeding coefficient *F*_ROH_ as the proportion of the genome in ROHs and considered individuals with *F*_ROH_ > 0.3536 to be selfed (Manichaikul *et al*., 2010), but reclassified any individuals with *F*_ROH_ > 0.3536 as non‐selfed if homozygous regions were dispersed in small segments rather than large contiguous stretches based on visual inspection of observed heterozygosity across the genome.

### Population genetic structure

For Pacific Northwest samples, we performed principal component analysis (PCA) using *EMU* v. 1.2.1 (Meisner *et al*., 2021). We filtered SNPs to a minimum minor allele frequency (MAF) of 0.01 and thinned to a minimum distance of 10 kbp before running *EMU* using 1000 iterations (*‐‐iter 1000*) and three eigenvectors for missing data estimation (*‐e 3*). For *Macrocystis* analyses including low‐depth California samples, we used the single read sampling PCA approach implemented in Angsd v.0.941 (Korneliussen *et al*., 2014) with BAM input, but the same set of sites (*−sites* flag) previously identified in the min1× dataset. We generated a covariance matrix in ANGSD using the flags *‐minMapQ 30 ‐minQ 30 ‐doMajorMinor 1 ‐doMaf 1 ‐doIBS 1 ‐doCov 1 ‐doCounts 1 ‐makeMatrix 1 ‐GL 1*. We then calculated eigenvalues and eigenvectors from the covariance matrix using the *eigen()* function in *R* v.4.4.2 (R Core Team, 2024).

We identified genetic clusters in the Pacific Northwest using fastStructure v.1.0 (Raj *et al*., 2014). We performed an initial 100 runs for each number of clusters (*K*) from *K* = 1 to 10. We selected the run with the highest likelihood for each *K*‐value and selected the optimal value using the script *chooseK.py* (distributed with *fastSTRUCTURE*). For both species, the optimal *K*‐value was 8. However, we selected *K* = 7 as our final result because the eighth cluster represented a locally bottlenecked population rather than a larger regional grouping in *Nereocystis*, and was geographically discontiguous and possibly a statistical artifact in *Macrocystis*.

We calculated population nucleotide diversity (π) from the global datasets including invariant sites for each species using pixy v.1.2.7.beta1 (Korunes & Samuk, 2021). We also used *pixy* to calculate pairwise genetic divergence (*d*_*XY*_; Nei & Li, 1979) between genetic clusters and between populations. To visualize patterns of isolation by distance, we plotted scaled genetic distance (*d*_*XY*_ – mean π) against pairwise ocean distance between populations (calculated using the *R* package gdistance v.1.6.5; Van Etten, 2017). The scaled genetic distance approximates the proportion of *d*_*XY*_ not attributable to within‐population genetic diversity. We calculated pairwise genetic differentiation (*F*_ST_; Weir & Cockerham, 1984) using hierfstat v.0.5–11 (Goudet, 2005) with minimum MAF 0.01 and thinned to 10 kbp. We also calculated the directionality index (ψ; Peter & Slatkin, 2013) in the Pacific Northwest for each species, filtered to minimum MAF of 0.05 and thinned to minimum of 10 kbp. ψ infers the direction of range expansions between populations, given shifts in allele frequencies that occur during founder events. We polarized alleles for ψ calculation following Bemmels *et al*. (2025) from alignments to outgroup genomes (Grigoriev *et al*., 2021; Phaeoexplorer Project, 2024) using bwa‐mem v.0.7.17‐r1188 (Li, 2013), SAMtools v.1.17 (Danecek *et al*., 2021), *HTSBox* v.r345 (Li, 2012), and scripts from Taylor *et al*. (2024). For further details, see Methods S1.

To further explore the relationship between *Macrocystis* from the Pacific Northwest and California, we constructed a phylogeny (Lewis, 2001; Kalyaanamoorthy *et al*., 2017) and assessed branch support (Guindon *et al*., 2010; Hoang *et al*., 2018) using Iq‐Tree v.2.3.6 (Minh *et al*., 2020) from SNPs thinned to 10‐kbp, retaining one individual per population. We tested for the possibility of gene flow between different geographic regions using four‐taxon Patterson's *D*‐statistics (Durand *et al*., 2011) inferred in Dsuite v.0.5 r53 (Malinsky *et al*., 2021). For further details, see Methods S1.

### Ecological niche modeling

To explore where climatically suitable habitat may have existed for kelp during the LGM, we constructed simple ecological niche models (ENMs) using MaxNet v.0.1.4 (Phillips *et al*., 2006, 2017) within the *R* package sdmtune v.1.3.1 (Vignali *et al*., 2020). We used occurrence records for both species from the Global Biodiversity Information Facility (GBIF.org, 2024a,b,c) and controlled for spatial bias using brown algae (Phaeophyceae) as background points to inform the model of assumed spatial bias in sampling effort of the target taxon (Phillips *et al*., 2009; Barber *et al*., 2021). We used environmental predictor variables characterizing temperature and salinity regimes from MARSPEC (Braconnot *et al*., 2007; Sbrocco & Barber, 2013; Sbrocco, 2014). For further details, see Methods S1.

### Ancestral recombination graphs

We constructed ARGs using Relate v.1.2.3 (Speidel *et al*., 2019, 2021). Full details of ARG construction are provided in Methods S1. In brief, we used datasets filtered to the Pacific Northwest (with SNPs polarized as described in the ‘Pairwise directionality index details’ section of Methods S1), removed selfed individuals (Mather *et al*., 2020), statistically phased and imputed missing data using Beagle v.5.5 (Browning *et al*., 2018, 2021), and estimated the required recombination map using FastEprr v.2.0 (Gao *et al*., 2016). We placed an initial prior on the mutation rate of 8.135 × 10^−10^ for both species, following Bemmels *et al*. (2025), and priors on haploid effective population size of 2 *n* = 200 000 and 20 000 for *Nereocystis* and *Macrocystis*, respectively. For rescaling results to absolute time in years, we assumed a generation time of 1 yr in the annual species *Nereocystis* and 2 yr in the perennial species *Macrocystis* based on qualitative interpretation of life history and ecological data (Dayton *et al*., 1984; Reed, 1987; Bell & Siegel, 2022), as outlined in Methods S1. We created initial ARG topologies in *Relate* (*‐‐mode all*) and then simultaneously re‐estimated branch lengths, effective population sizes (*N*_e_), and mutation rates using the Relate script *EstimatePopulationSize.sh*. We also repeated ARG reconstruction for *Macrocystis* using the global dataset thinned to one population per genetic cluster from the Pacific Northwest and all populations from Chile and California.

We estimated the demographic history of each population and the timing of population splits using the output from *EstimatePopulationSize.sh*. We calculated *N*_e_ in each time bin as 0.5/*r*, where *r* is the haploid coalescence rate (Speidel *et al*., 2019), and took the mean across all chromosomes. We also calculated the relative cross‐coalescence rate (rCCR) as the coalescence rate between populations divided by the mean coalescence rate within populations (Schiffels & Wang, 2020). To prevent rare extreme outliers (that arise when comparing ratios of very small numbers) from distorting overall patterns, we arbitrarily set the upper limit on rCCR for each chromosome to 2, before averaging across chromosomes. We estimated divergence time between populations as the midpoint of the most recent time bin at which the rCCR first rose above 0.5 (Schiffels & Wang, 2020).

### Inferred ancestral locations

We used *Spacetrees* (Osmond & Coop, 2024) to infer the geographic locations of genetic ancestors through time from the ARGs for the Pacific Northwest. *Spacetrees* spatially models the Brownian motion of ancestors down trees in an ARG. To reduce correlations between trees, we first thinned to every 500^th^ tree, resulting in 844 trees for *Nereocystis*, and to every 100^th^ tree, resulting in 887 trees for *Macrocystis*. To account for uncertainty in branch length estimates, each of these trees was sampled 100 times (using *Relate*'s *SampleBranchLengths.sh* script). Ancestor locations were then inferred using *Spacetrees*’ best linear unbiased predictor (BLUP) method, which averages the most likely location over samples of each tree, as this method is fast and robust to outlier branch length estimates. For visualization, we calculated mean ancestor locations across trees for each individual in the entire dataset (all Pacific Northwest samples) and for each genetic cluster. For each genetic cluster, we also calculated spatial contour lines containing 90% of all inferred ancestor locations using the *contour()* function in *R* v.4.4.2 (R Core Team, 2024) from a kernel density of 100 × 100 pixels generated with the *kde2d()* function in the *R* package mass (Venables & Ripley, 2002).

To explore the robustness of the *Spacetrees* results, we conducted two additional tests. Firstly, we performed a randomization analysis by randomly permuting the geographic location of each individual and rerunning *Spacetrees*. This test was performed only once as a means of qualitatively visualizing model results when there is no spatial signal in the ARG. Secondly, we performed a leave‐one‐out analysis by removing a single population at a time and rerunning *Spacetrees*. This analysis was performed once per population (from populations north of Vancouver Island only) as a means of visualizing uncertainty in mean ancestor locations and testing whether particular northern populations could be responsible for pulling mean ancestor locations northward when using the full dataset.

## Results

### Population genetic structure

**Fig. 1:**
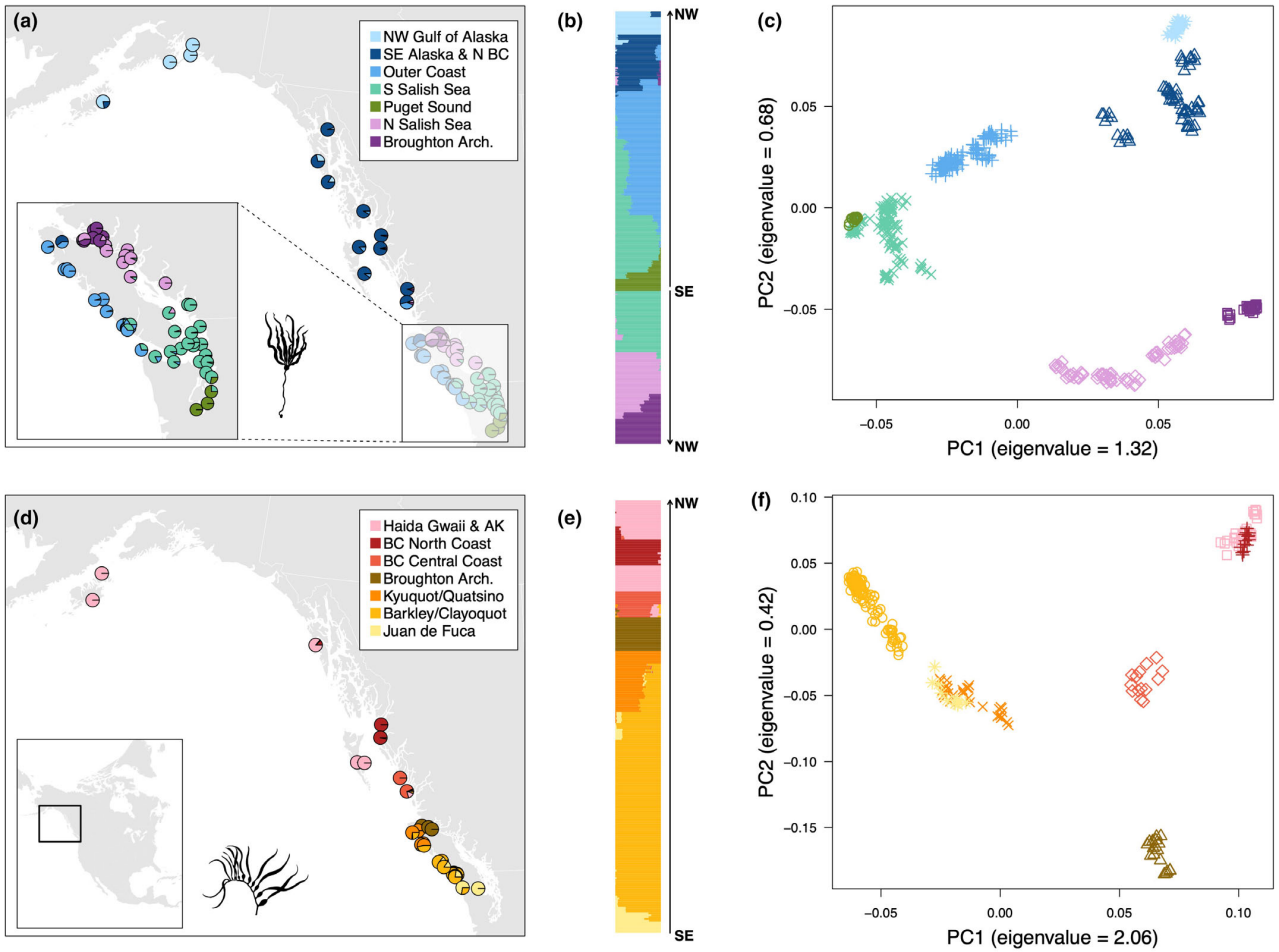
Population genetic structure in the Pacific Northwest for (a–c) *Nereocystis* and (d–f) *Macrocystis*. (a, d) Genetic clusters identified in faststructure, with pie charts showing the proportion of each population's ancestry in each genetic cluster. AK, Alaska; arch., archipelago. (b, e) Ancestry in genetic clusters, with each horizontal line representing one individual. Individuals are arranged geographically from southeast (SE) to northwest (NW). (c, f) PCA of genetic variation, with each point representing an individual and each genetic cluster represented by a different symbol and color.

**Fig. 2:**
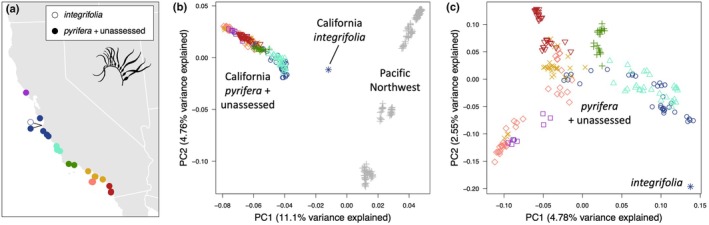
Population genetic structure of *Macrocystis* in California. (a) Map of populations showing colors used to represent individuals in (b, c). Open circle, *integrifolia* morph; closed circles, *pyrifera* morph and populations for which morph identity was unassessed. (b) PCA of genetic variation in California and the Pacific Northwest. Note that the Pacific Northwest includes both *pyrifera* (Alaska) and unassessed but presumed *integrifolia* (southern BC and Washington) morphs. (c) PCA of genetic variation in California only.

**Fig. 3:**
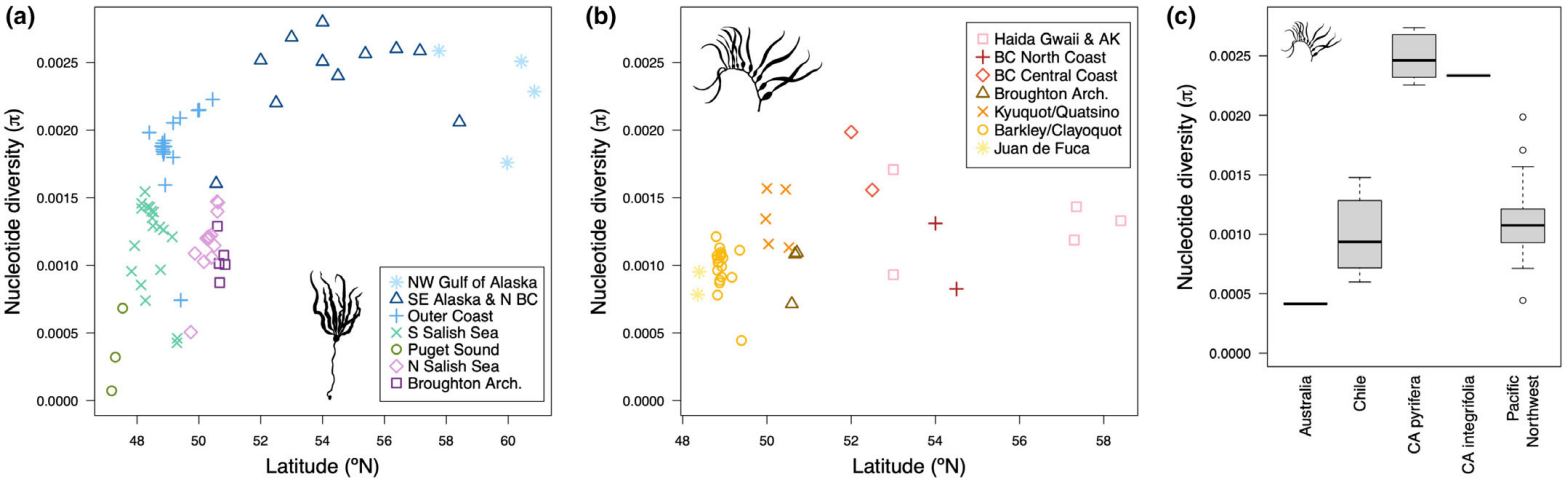
Geographic patterns of genetic diversity. (a, b) Relationship between population nucleotide diversity (π) and latitude (°N) in the Pacific Northwest for (a) *Nereocystis* and (b) *Macrocystis*. (c) Variation in π among *Macrocystis* populations from different global regions. CA, California. Boxplot details: shaded box area, interquartile range; horizontal line, median; whiskers, most extreme data point within 1.5× the interquartile range from the box; outliers, data points > 1.5× the interquartile range from the box.

Genetic clusters identified in fastStructure and PCAs of genetic variation corresponded closely with geography and identified a close relationship between northern BC and newly sequenced Alaska samples in both species (Fig. 1). Among North American *Macrocystis*, the first principal component (PC) axis of variation separated Pacific Northwest from Californian individuals, with the single verified *integrifolia* individual from California falling in an intermediate position between Pacific Northwest and other Californian individuals (Fig. 2a,b). Verified *pyrifera* from Alaska clustered with other Pacific Northwest samples (unassessed but presumably mostly *integrifolia*) rather than with verified *pyrifera* from California. Within California, genetic variation among *pyrifera* and unassessed populations primarily followed a latitudinal gradient (Fig. 2c), though despite the use of a PCA method sampling only a single read per site as an attempt to reduce read‐depth biases, low‐coverage samples were drawn toward the origin (Fig. S2) suggesting a statistical artifact. Separate from this artifact, the northernmost population from Bodega Bay (purple) unexpectedly clustered with the offshore Channel Islands (pink). Within the Pacific Northwest, genetic diversity (π) was highest in north‐central BC for both species (Fig. 3a,b). Genetic diversity was positively correlated with mean depth of coverage across populations in *Nereocystis* (*P* = 0.0076; Fig. S3a) but not *Macrocystis* (Fig. S3b). This correlation in *Nereocystis* is unlikely to reflect genotyping bias as it is driven by newly sequenced high‐diversity populations from Alaska (> 55° N) that also have high coverage (Fig. S3c), yet higher‐coverage Southeast Alaska populations do not show elevated diversity relative to lower‐coverage northern BC populations from the same genetic cluster (blue triangles; Fig. S3e) and peak diversity occurs in northern BC and declines both northward and southward regardless of coverage (Fig. S3e), suggesting that geography and not sequencing depth drives variation in genetic diversity. Globally, the highest *Macrocystis* diversity was found in California and was similar for both morphs (Fig. 3c), with intermediate diversity in the Pacific Northwest and Chile and lowest diversity in Australia.

Genetic divergence (*d*_*XY*_) among genetic clusters and regions largely corresponded to geography, with more distant populations exhibiting higher *d*_*XY*_ (Figs S4, S5). In southern BC and Washington, the relationship between scaled genetic distance and geographic distance was similar whether populations belonged to the same or different genetic clusters (Fig. S5), suggesting that clusters partly reflect isolation by distance (also supported by the frequent detection of genetically intermediate populations; Fig. 1). However, within genetic clusters covering northern BC and Alaska, scaled genetic distance was lower than expected based on geographic distance for both species, suggesting higher genetic connectivity or more recent expansion across northern regions. Among global *Macrocystis*, *d*_*XY*_ was much higher between hemispheres than within hemispheres. The *d*_*XY*_ between southern Pacific Northwest individuals and California *integrifolia* was lower than between southern Pacific Northwest and California *pyrifera*, or between the two morphs within *California*. Genetic differentiation (*F*_ST_) did not show a consistent geographic pattern as it appeared strongly impacted by π, with low‐diversity populations exhibiting high *F*_ST_ (Fig. S4).

**Fig. 4:**
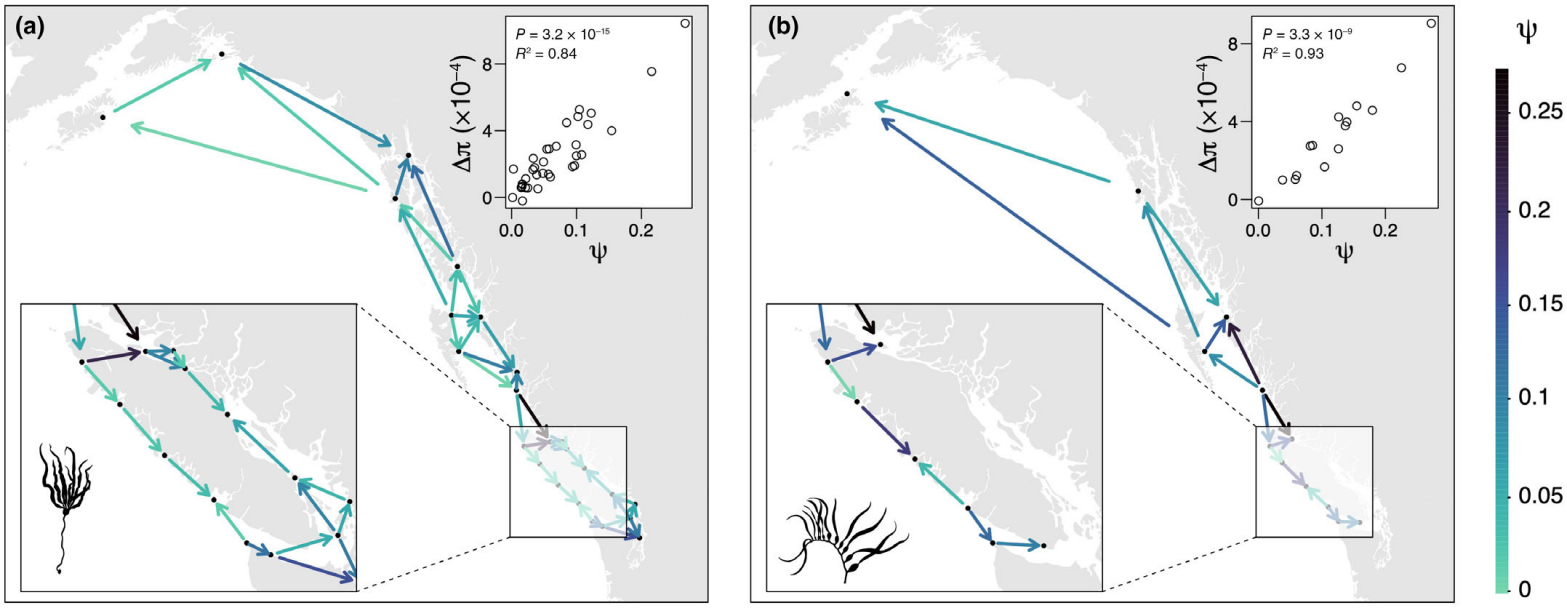
Directionality index (ψ) between pairs of populations of (a) *Nereocystis* and (b) *Macrocystis*. Arrows point in the inferred direction of range expansion and are colored according to the magnitude of ψ, with higher ψ indicating a stronger signal of range expansion. Inset plots show the relationship between the population pairwise difference in nucleotide diversity (Δπ) and ψ.

Pairwise directionality indices (ψ) revealed similar inferred directions of postglacial range expansion in both species (Fig. 4), beginning in Haida Gwaii for *Nereocystis* and the central coast of BC for *Macrocystis* and proceeding in both a northerly and southerly direction. As expected for recent range expansions (Kemppainen *et al*., 2024), pairwise ψ was highly correlated with the difference in nucleotide diversity (Δπ) between populations (Fig. 4).

### Macrocystis global phylogeny and gene flow

The Iq‐Tree phylogeny of *Macrocystis* revealed a deep split between northern and southern hemispheres (Fig. S6a). Within North America, Southern California *pyrifera* occupied the earliest diverging lineages. Northern California *pyrifera* was sister to a clade containing northern California *integrifolia* and the Pacific Northwest. Alaskan individuals (all verified *pyrifera*) did not group with Californian *pyrifera* but rather belonged to the clade containing other Pacific Northwest individuals. Support for clades that distinguished major global geographic regions was always high (SH‐aLRT ≥ 80% and ultrafast bootstrap support ≥ 95%), but branching patterns within smaller geographic regions were often poorly resolved (Fig. S6a).

Statistically significant signatures of gene flow were inferred in *Dsuite* between *integrifolia* and *pyrifera* morphs in northern California (box 1 in Fig. S6c) as well as between California *integrifolia* and southern Pacific Northwest populations (box 2 in Fig. S6c). Numerous other instances of gene flow were also inferred, mostly between geographically adjacent populations. Strong signals of gene flow between central BC (MP‐CC‐01 and MP‐PH‐01) and all individual southern BC populations may reflect the uncertain phylogenetic placement of central BC (Fig. S6a) or a non‐tree‐like relationship among populations from this region.

### Ecological niche models

Spatial biases in sampling effort were qualitatively similar between the focal species and brown algae that were used as background points to control this bias (Fig. S7). For both species, the same three final predictor variables were retained: mean annual sea surface salinity (SSS), sea surface temperature (SST) of the warmest ice‐free month, and annual range in SST (Table S3). Predictive performance (Table S4) was moderate in terms of testing area under the receiver operating characteristic curve (AUC: 0.78–0.80; moderate AUC category: 0.7–0.9, Smith *et al*., 2021) and fair to moderate in terms of true skill statistic (TSS: 0.52–0.54; fair to moderate TSS category: 0.2–0.6, Smith *et al*., 2021).

The predicted distributions of both species in the current time period matched known distributions well (Figs S7, S8), especially in *Nereocystis*. In *Macrocystis*, the model overpredicts presence (though with only marginal suitability) in some areas where it does not occur, including inner waterways (e.g. the inner Salish Sea and innermost Southeast Alaska) and west of Kodiak Island (Fig. S8b). The ENMs projected to the LGM ensemble model excluding CCSM3 (see Methods S1) predicted marginally suitable habitat along the coastline of Alaska and BC in *Nereocystis* (Fig. S9a). In *Macrocystis*, the LGM ensemble projections predicted almost complete absence from this region (Fig. S9b). However, when projected to the LGM CCSM3 climate model, the coastlines of Alaska and BC showed many areas of moderate habitat suitability in ice‐free areas for *Nereocystis* and moderate to low suitability for *Macrocystis* (Fig. S10).

### Demographic and geographic inferences with ARGs

**Fig. 5:**
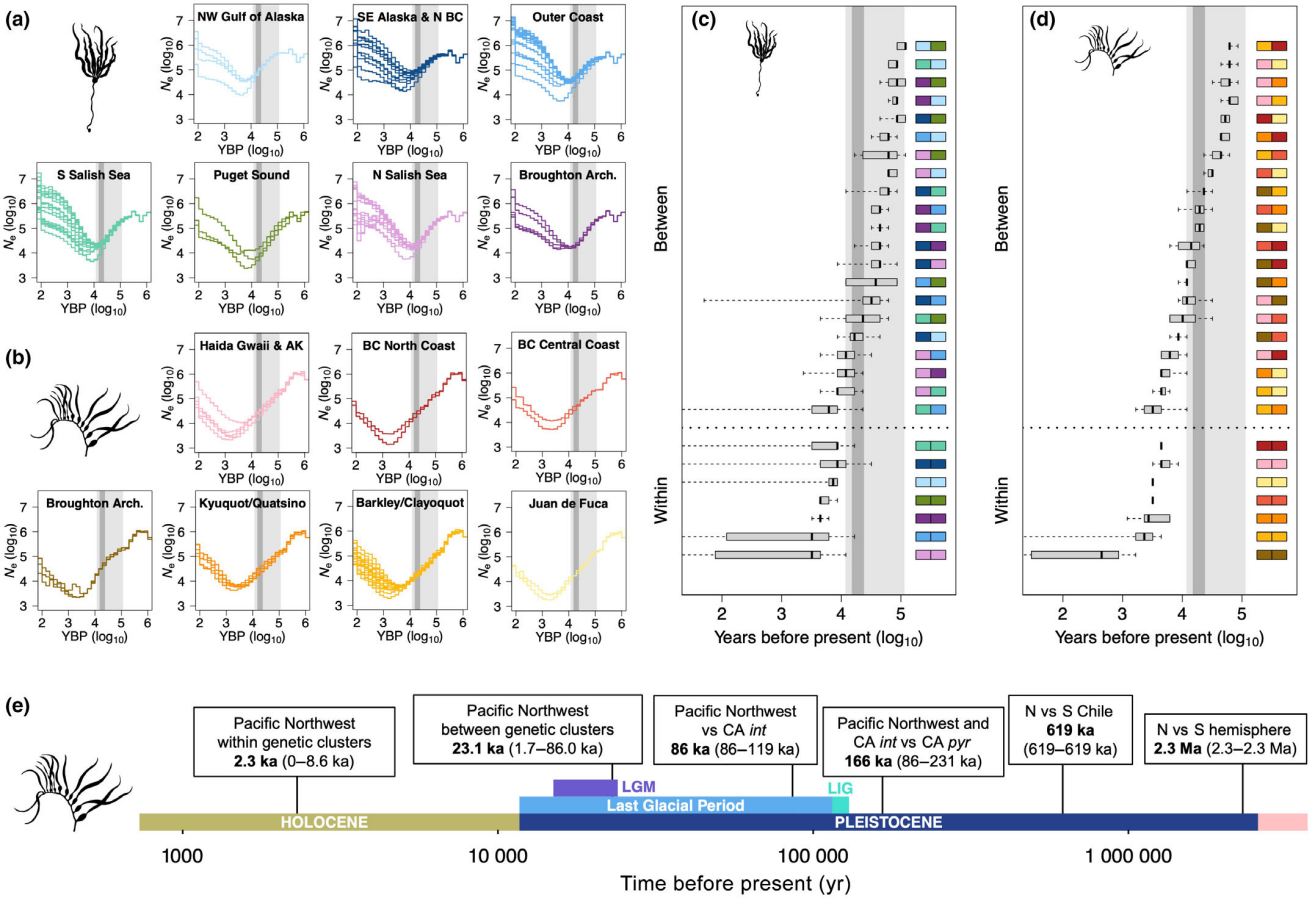
Population demographic events inferred from ARGs. (a, b) Changes in effective population size (*N*_e_) over time in populations of (a) *Nereocystis* and (b) *Macrocystis*, with populations grouped by genetic cluster. Light and dark gray horizontal bands show the timing of the Last Glacial Period and the local Last Glacial Maximum (LGM), respectively. AK, Alaska; arch., archipelago; YBP, years before present. (c, d) Inferred population split times between pairs of populations within and between genetic clusters for (c) *Nereocystis* and (d) *Macrocystis* in the Pacific Northwest. Pairs of genetic clusters are color‐coded as in (a) and shown on the right of the plot for each boxplot. Boxplots show the distribution of split times among all possible pairs of populations corresponding to each pair of genetic clusters, with whiskers extending to minimum and maximum values. See also Supporting Information Table S5 for a textual representation. (e) Timeline of inferred population split times between pairs of *Macrocystis* populations from different global regions, with median split times among all possible population pairs in bold and the corresponding range in parentheses. If the minimum and maximum of the range are identical, then all possible population pairs were inferred to have diverged in the same time bin. CA, California; int, *integrifolia* morph; pyr, *pyrifera* morph; LIG, Last Interglacial. Boxplot details: shaded box area, interquartile range; vertical line, median; whiskers, most extreme data points.

Effective population sizes (*N*_e_) inferred in *Relate* suggested late Pleistocene to Holocene bottlenecks in all populations of both species (Fig. 5a,b). In *Nereocystis*, bottlenecks typically reached minimum size near the Pleistocene–Holocene transition (11.7 ka; Fig. 5a). In *Macrocystis*, bottlenecks were more severe and more recent, with minima generally occurring between 10 and 1 ka. Divergence times among populations within the Pacific Northwest were similar in both species (Fig. 5c,d). Median divergence times (Table S5) between population pairs within the same genetic cluster dated to the Holocene (*Nereocystis*: 3.2–8.6 ka; *Macrocystis*: 0.4–4.4 ka), while those between different genetic clusters were often substantially older (*Nereocystis*: 6.1–119.5 ka; *Macrocystis*: 3.2–61.9 ka).

In *Macrocystis*, divergence times between populations from different global regions all dated to the Pleistocene (Fig. 5e). Pacific Northwest individuals and California *integrifolia* were estimated to have diverged at 86 ka (range among different population pairs: 86–119 ka), more recently than the divergence between Pacific Northwest and California *pyrifera* (166 ka; range: 119–231 ka) or between the two morphs within California (166 ka; range: 86–231 ka). Northern and southern Chilean populations diverged at 619 ka, while the Northern and Southern Hemisphere diverged at 2.3 Ma (with no range given for either estimate, as the 50% rCCR corresponded to the same time bin for all pairs of populations).

**Fig. 6:**
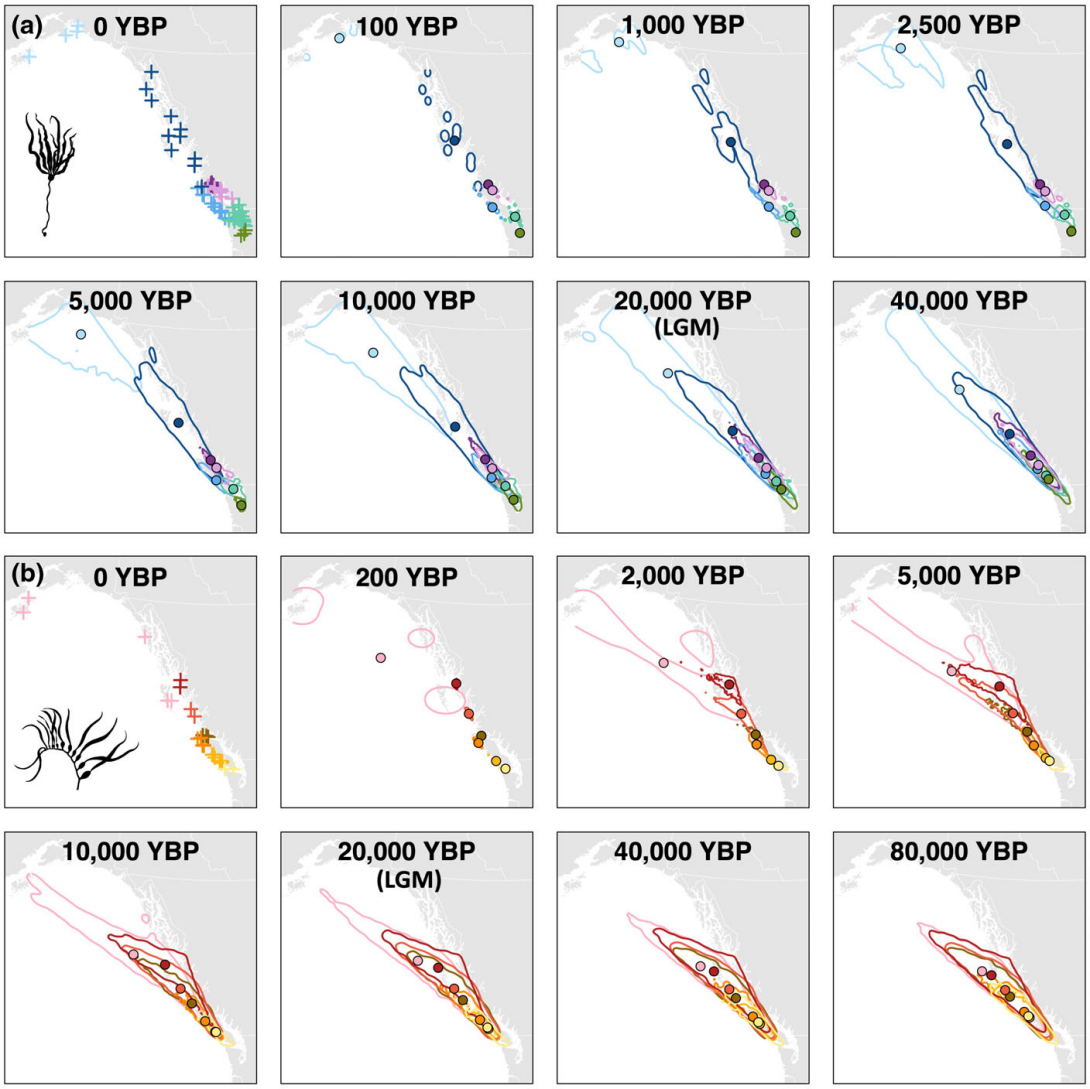
Geographic locations of ancestors through time inferred from ARGs using *Spacetrees* for (a) *Nereocystis* and (b) *Macrocystis*, visualized for each genetic cluster. The first panel at 0 yr before present (YBP) depicts the sampling locations of each population, with different colors representing different genetic clusters. In subsequent panels, lines represent contours enclosing 90% of all ancestor locations for each genetic cluster, and circles represent the mean ancestor latitude and longitude. LGM, Last Glacial Maximum.

The mean ancestor location inferred in *Spacetrees* toward which all populations collapse if allowed to coalesce toward completion (i.e. 10^6^ generations before present) is in southern Haida Gwaii for both species. This location should not be interpreted as the true mean ancestor location at 10^6^ generations because *Spacetrees* cannot accurately infer ancestor locations across multiple glacial cycles of range expansion and contraction (Osmond & Coop, 2024). However, the fact that it is substantially north of the mean latitude and longitude of sampled populations in the present day (northern Vancouver Island for both species) confirms that there is a spatial signal in the ARGs. At shallower time scales, inferred locations of genetic ancestors for each genetic cluster showed both species increasingly converging toward central and northern BC backward in time (Fig. 6). By contrast, the randomization test in which spatial signal was removed showed all genetic clusters quickly (≤ 100 generations) converging toward the mean sampling location (Fig. S11), further highlighting how the main results reflect spatial and temporal signal in the ARGs.

The broad distribution of inferred ancestor locations at 20 ka for each genetic cluster highlights uncertainty about precise ancestor locations at the LGM. In addition, genetic coalescence may substantially predate spatial convergence of lineages, creating a lag in *Spacetrees* between arrival at a true ancestral population location and genetically inferred locations at that time. Thus, the generations before the LGM may help clarify precise refugial locations. In *Nereocystis*, mean ancestor locations from 20 to 40 ka for all, but the northernmost genetic cluster, converge toward two separate locations: northern Vancouver Island and southern Haida Gwaii (Fig. 6a). The northernmost cluster shows a separate far‐northern location. In *Macrocystis*, the mean ancestor locations from 20 to 80 ka converge toward three locations: southern Haida Gwaii, the central coast of BC, and northern Vancouver Island (Fig. 6b). The leave‐one‐out analysis revealed further uncertainty in mean ancestor locations, especially in *Macrocystis*, where removing a small portion of the data could cause modest shifts in inferred ancestor locations (Fig. S12). However, in both species, the inferred ancestor locations always converged toward the same general region of north‐central BC (well north of the mean sample location), and convergence toward three distinct areas was always observed in *Macrocystis*.

## Discussion

Canopy‐forming kelp forests likely persisted throughout the LGM in northern refugia along ice‐free margins of the glaciated Pacific coastline of North America. Range expansion from these refugia gave rise to contemporary populations of *Nereocystis* and *Macrocystis* from Washington to Alaska. ARG‐based inference of ancestral locations using *Spacetrees* agreed with qualitative interpretations of genetic diversity, pairwise directionality indices, and simple ENMs, all of which highlighted the central to northern coast of BC as a likely refugial region. In addition, *Spacetrees* added increased resolution and suggested there may have been multiple refugia within this region, as ancestor locations of different populations converged toward either southern Haida Gwaii, the central coast of BC, or northern Vancouver Island. These regions were previously inferred from geologic evidence to have been continuously ice‐free. Our results highlight the power of ARG‐based models to geographically locate ancestors through time and reconstruct the origins of range expansions. Despite the inference of northern refugia, hints of gene flow in *Macrocystis* between the southern Pacific Northwest and California (and between morphs within California) require further study.

### Kelp persistence in northern refugia

Descriptive phylogeographic approaches and the *Spacetrees* model agreed in identifying northern refugia along glacial margins, providing strong support for survival of both species in ice‐free pockets of central and northern BC. Supporting evidence from descriptive approaches includes: higher genetic diversity in this region than elsewhere in the Pacific Northwest (Fig. 3); pairwise directionality index (ψ) values (Fig. 4) suggesting that range expansion proceeded from this region in both a northerly and southerly direction; and simple ENMs suggesting that low‐ to moderate‐quality habitat was available in this region (Figs S9, S10).

However, although these lines of evidence are strongly compatible with northern refugia, qualitative interpretation of genetic diversity and ψ does not provide definitive evidence of refugial locations for several reasons. High genetic diversity is expected in refugial locations due to loss of genetic diversity from successive founder events during postglacial expansion (Hewitt, 2000), but may also reflect secondary contact between lineages expanding from other areas (Petit *et al*., 2003) or suggest large contemporary population size, which varies widely and is highly correlated with π in both species (Bemmels *et al*., 2025). In addition, the core‐periphery hypothesis predicts that genetic diversity should be higher in the middle of a species' range and lower at the edges due to decreased gene flow and stronger genetic drift in small populations at the range margins (Eckert *et al*., 2008). Core‐periphery dynamics may also severely bias ψ (Kemppainen *et al*., 2024). As Washington to Alaska could be considered a contiguous biogeographic unit given low abundance farther south in Oregon (Fig. S7), the north coast of BC would represent the geographic core of this unit and correspond to the area of expected highest diversity. However, the core‐periphery hypothesis has limited support in other marine taxa (Cárcamo *et al*., 2025) and would require that the genetic signal of postglacial range expansion has been totally erased, which seems unlikely given the short time that has elapsed since the LGM and strong genetic structure (Figs 1, S4). Finally, as expected immediately following range expansion (Kemppainen *et al*., 2024), ψ was highly correlated with pairwise Δπ (inset plots in Fig. 4). Thus, ψ cannot be taken as an independent source of information to strengthen confidence in interpretations of π, but instead provides a visualization of the shared signal inherent in both statistics.

Given the difficulties in qualitative interpretation of genetic patterns, the *Spacetrees* analysis that leveraged spatial signatures from > 800 local trees across the genome to infer the coordinates of genetic ancestors provided a powerful tool to locate refugia and to visualize key features of postglacial range expansion. *Spacetrees* showed increasing geographic convergence of ancestors backward in time toward north‐central BC in both species (Fig. 6). At 20 ka, there is a broad spatial distribution of inferred ancestor locations (90% contour lines; Fig. 6). However, as coalescence predates the time of population splitting in the absence of gene flow (Edwards & Beerli, 2000), some lineages that expanded from the same refugium may have coalesced far earlier than 20 ka. It is thus relevant to consider trajectories of ancestor locations before 20 ka as coalescence continues. At 40 ka in *Nereocystis* and 40–80 ka in *Macrocystis*, mean ancestral locations were mostly distributed from northern Vancouver Island to Haida Gwaii (Fig. 6), highlighting the strong signal of range expansion from this region. Importantly, there was no evidence of convergence toward the south in either species. In addition to looking farther back in time than 20 ka, the absolute timing of coalescence events (and absolute *N*_e_) must be interpreted with caution, as their scaling depends on the mutation rate, which we based on two filamentous brown algae that are only distantly related to the study species (Krasovec *et al*., 2023; Bemmels *et al*., 2025). The timing of inferred bottlenecks at or immediately after the LGM (Fig. 5a,b) aligns with expectations based on historical events, suggesting that our temporal scaling is largely accurate, but nonetheless, all absolute dates (Figs 5, 6) should be considered approximate.

### A region of multiple refugia

In addition to confirming the likely existence of northern refugia in north‐central BC, ARG‐based analyses point to the potential existence of multiple refugia within this region. Evidence of multiple refugia is clearest in *Macrocystis*, where the mean ancestor locations for the seven genetic clusters converge toward three separate geographic locations (Fig. 6b): southern Haida Gwaii, the central coast of BC, and northern Vancouver Island. This convergence to three locations is not transitory, as might be expected if it reflected an intermediate state of migration between a refugial and non‐refugial scenario, but clearly persists from 20 to 80 ka (Fig. 6b). Furthermore, population divergence times in *Macrocystis* are more recent than the LGM between populations that converge toward the same location, but mostly overlap with or are more ancient than the LGM between populations that converge toward different locations (Fig. 5d; Table S5), suggesting that there was more than one refugium present at the LGM. Additionally, bottlenecks with minimal *N*_e_ in the thousands to tens of thousands (Fig. 5b) suggest at least moderately sized refugia and are not compatible with collapse of all populations into a single, extremely small population. Finally, the division of populations into three groups along the first PC axis (Fig. 1f) corresponds precisely to the division of populations into three ancestral locations in *Spacetrees* (Fig. 6b), suggesting that contemporary genetic structure may have its origins in the three distinct LGM refugia.

In *Nereocystis*, the number of refugia is more difficult to determine. Ancestor locations show less spatial convergence and lower stability over time, but spatial convergence from 20 to 40 ka (Fig. 6a) suggests at least two main refugia in southern Haida Gwaii and northern Vancouver Island. In addition, divergence times between northern and southern populations are more ancient than the LGM (Fig. 5c; Table S5), as expected if northern and southern populations are derived from separate refugia. The ancestors of the northernmost (light blue) cluster are located at latitudes equivalent to Southeast Alaska from 20 to 40 ka (Fig. 6a), but it is unlikely there was a separate refugium in Alaska because the inferred divergence time between the two northernmost clusters dates to the LGM (Fig. 5c) and the 90% contour line for this cluster at 20 ka includes southern areas, indicating some coalescence with southern populations. Instead, we speculate that this genetic cluster may have originated through long‐distance migration across the Gulf of Alaska, which the Brownian motion dispersal model in *Spacetrees* is not designed to accommodate (Osmond & Coop, 2024).

Remarkably, the inferred refugial locations correspond almost exactly with geological reconstructions, suggesting several disjunct ice‐free areas of coastline separated by glacial lobes (Shaw *et al*., 2020; Mann & Gaglioti, 2024). These ice‐free areas were located off the coast of southeastern Haida Gwaii (the ‘Hecate refugium’), the central coast of BC, and northern Vancouver Island, precisely where *Spacetrees* inferred refugia for *Macrocystis* and most populations of *Nereocystis*. As the spatial spread of inferred ancestral locations was extremely broad at all time periods (Fig. 6), suggesting high uncertainty, and the leave‐one‐out analysis identified low precision regarding exact refugial latitudes (Fig. S12), we cannot rule out that this result could have been coincidence. Nonetheless, ENM projections largely suggest that LGM persistence in these ice‐free regions is plausible (Figs S9, S10). Although suitable habitat was not predicted in this region for *Macrocystis* in the ensemble climate model excluding CCSM3 (Fig. S9b), the ensemble model does not correctly account for increased ocean salinity due to freshwater glacier formation and a drop in sea level during the LGM (Sbrocco, 2014). As salinity was an important predictor of habitat suitability for *Macrocystis* (Table S3), the CCSM3 model that corrects for increased salinity may be most appropriate for this species. Additional areas of predicted suitability exist in southern Vancouver Island and Southeast Alaska but were unlikely to have contained refugia for our study species. Southern Vancouver Island experienced an extremely late local LGM (Mann & Hamilton, 1995; Mann & Gaglioti, 2024) and was eventually overrun by glaciers, which is not reflected in the glacial reconstruction for the global LGM (Gillespie *et al*., 2004) depicted in Figs S9 and S10. Meanwhile, seasonal sea ice may have covered the entire coast of Southeast Alaska during the LGM (Praetorius *et al*., 2023; Mann & Gaglioti, 2024) suggesting that this region presented very different winter environmental conditions than either species experiences today, which casts doubt on inferred habitat suitability north of Haida Gwaii.

**Fig. 7:**
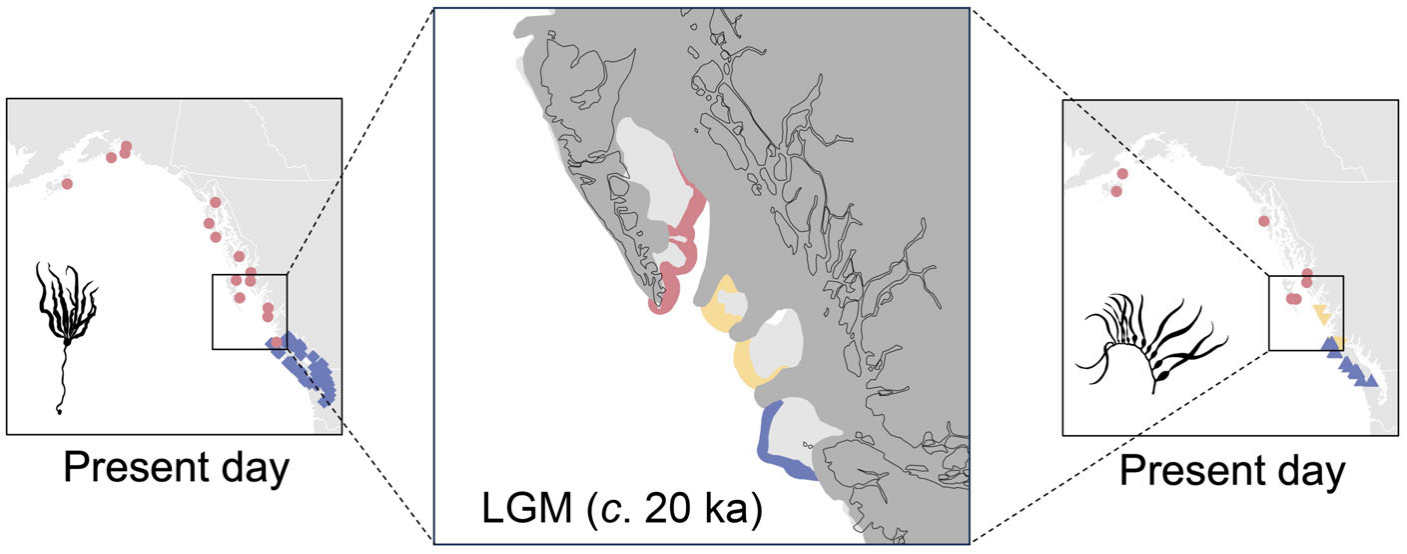
Hypothesized refugial scenario during the Last Glacial Maximum (LGM), showing a qualitatively interpreted summary of all results, especially Fig. 6. The central panel shows hypothesized refugia off the coasts of southern Haida Gwaii (red), central British Columbia (yellow), and northern Vancouver Island (blue), with populations sampled in the present day that originated primarily from these refugia in corresponding colors for *Nereocystis* (left) and *Macrocystis* (right). The central panel depicts LGM glaciers (dark gray), exposed continental shelf (light gray), and the modern coastline (black lines). The location of glaciers and continental shelf is a cartoon representation redrawn by hand from Shaw *et al*. (2020).

The agreement between genomic, geological, and climatological (i.e. ENM) evidence allows us to reconstruct a hypothesized refugial scenario for both species (Fig. 7). In *Macrocystis*, convergence of ancestral locations (Fig. 6) toward southern Haida Gwaii, the BC Central Coast, and northern Vancouver Island aligns with the three major ice‐free areas of north‐central BC (Shaw *et al*., 2020), which are all areas where LGM climates may have been amenable to persistence of both species (Fig. S10). We hypothesize that each of these three areas may have hosted *Macrocystis* refugia during the LGM, separated by glacial lobes (Fig. 7). The situation is similar in *Nereocystis*, except that in *Spacetrees* (Fig. 6), most genetic clusters converge toward either southern Haida Gwaii (red, Fig. 7) or northern Vancouver Island (blue, Fig. 7), suggesting these may have been the primary two refugia for *Nereocystis*. However, due to high uncertainty, we cannot rule out that the BC Central Coast (yellow, Fig. 7) could also have hosted refugia or that north‐central BC may have effectively formed a single refugial region connected by high gene flow in *Nereocystis*. We also cannot rule out the possibility of additional, undetected refugia located farther north (e.g. Alaska) that did not substantially contribute to contemporary populations of either species.

### Global relationships in *Macrocystis*

*Macrocystis* populations from different global regions have a long and independent history. Divergence times from *Relate* (Fig. 5c) suggest that the earliest split occurred between the northern and southern hemispheres at 2.3 Ma, near the onset of the Pleistocene (2.58 Ma). The late Pliocene to early Pleistocene was a period of major transition, with Pleistocene oceans characterized by colder temperatures and strengthened cold‐water upwelling in the Pacific (Filippelli & Flores, 2009). Closure of the Isthmus of Panama *c*. 2.75 Ma also reorganized ocean circulation and resulted in increased nutrient concentrations at low latitudes of the eastern Pacific (Schneider & Schmittner, 2006). We speculate that these global changes could have created the conditions necessary for dispersal to the South Pacific from the North Pacific, where *Macrocystis* likely originated (Coyer *et al*., 2001; Starko *et al*., 2019; Assis *et al*., 2023). Dispersal may have been most likely during a glacial period when distributions of temperate seaweed species shifted toward lower latitudes (Song *et al*., 2021) and perhaps could have made use of (sub)tropical islands as stepping stones (Assis *et al*., 2018). Deep divergences also exist within the Southern Hemisphere (619 ka split between Northern and Southern Chile), in agreement with previous findings (Gonzalez *et al*., 2023; Lindstrom, 2023).

Within North America, divergences between morphs and between California and the Pacific Northwest are more recent but substantially predate the LGM (Fig. 5c). However, several lines of evidence hint that there may have been some genetic contact between California and the Pacific Northwest after they initially diverged, including (1) the intermediate phylogenetic (Fig. S6a) and PCA (Fig. 2) position of California *integrifolia* between California *pyrifera* and all Pacific Northwest samples; (2) reduced *d*_*XY*_ between California *integrifolia* and southern but not northern BC (Fig. S4); and (3) a signal of gene flow between California *integrifolia* and southern BC populations (box 2 in Fig. S6c).

Interpreting the phylogenetic placement of California *integrifolia* and divergence times with other populations at face value would suggest that California *integrifolia* and all Pacific Northwest populations (including Alaskan *pyrifera*) share a single origin, and there has been subsequent gene flow between California *integrifolia* and southern BC (Hypothesis 1; Fig. S6b). Alternatively, it is also plausible that California *integrifolia* is sister to southern BC but has subsequently introgressed with northern California *pyrifera* (Hypothesis 2; Fig. S6b), causing an intermediate phylogenetic position (Fig. S6a). This scenario may have occurred, for example, if California *integrifolia* is descended from long‐distance migrants from southern BC or Washington. We are unable to distinguish these two scenarios, and the relationship between California and the Pacific Northwest requires further study, including expanded sampling of *integrifolia* from multiple locations in California. Relationships among California *pyrifera* also require clarification, given the unexpected clustering of a northern Californian population from Bodega Bay (purple) with the distant Channel Islands (pink) in the PCA (Fig. 2), possibly suggesting long‐distance migration and complex but undocumented genetic structure within California (Alberto *et al*., 2010; Johansson *et al*., 2015; Assis *et al*., 2023; Gonzalez *et al*., 2023). Furthermore, the close clustering of *pyrifera* morphs from Alaska with presumed *integrifolia* morphs from southern BC and Washington, rather than with other *pyrifera* morphs from California (Figs 2b, S6a), highlights how examining multiple geographic transition zones between morphs (i.e. both California and Alaska) will be required to fully understand the genetic basis of morph identity in North America (Gonzalez *et al*., 2023; Gonzalez & Raimondi, 2024) or possible lack thereof.

### Implications and conclusions

As canopy‐forming kelps are foundation species that create upright, spatially structured habitats upon which numerous species rely (Teagle *et al*., 2017; Wernberg *et al*., 2019), their inferred persistence in northern refugia suggests that biodiverse kelp forest ecosystems could plausibly have survived at high latitudes of the North American Pacific Coast throughout the LGM. The concordance between species (Fig. 7), despite *Nereocystis* being more widely distributed in the Pacific Northwest (Fig. S7) and more of an ecological generalist than *Macrocystis* (Druehl, 1978), further hints that a variety of marine species with various ecological niches may have been capable of surviving in northern refugia. However, further study of diverse kelp‐forest‐associated organisms is needed to confirm this hypothesis, as co‐distributed species often exhibit species‐specific phylogeographic histories (Stewart *et al*., 2010; Papadopoulou & Knowles, 2015) and communities with no close modern analogues were common in the past (Jackson & Williams, 2004; Graham, 2005). In Southern California, kelp forest productivity is believed to have fluctuated since the LGM, peaking during the mid‐Holocene (Graham *et al*., 2010). It is thus possible that the LGM kelp forests of north‐central BC may have substantially differed from modern kelp forests in their species assemblages or ecosystem functions. The assumption that biodiverse kelp forests were present along northern Pacific coastlines during deglaciation in the millennia following the LGM has also led to the hypothesis that humans may have first migrated from Asia to North America by a coastal route, taking advantage of the abundant resources provided by kelp forests (Erlandson *et al*., 2007, 2015; Braje *et al*., 2017). Our results are compatible with this hypothesis, as they suggest that canopy‐forming kelps were already present during the LGM in northern refugia, from which they could have rapidly expanded along the proposed human migration route as glaciers melted. Nonetheless, assessing whether early humans used the marine resources associated with *Nereocystis* or *Macrocystis* forests – or even migrated by a coastal route at all – remains outside the scope of this manuscript.

In summary, our work extends northern refugia paradigms initially developed for terrestrial species to marine plants and adds to a growing body of research showing that temperate terrestrial and marine species often survived much closer to glacial margins than initially suspected (Stewart & Lister, 2001; Jacobs *et al*., 2004; Provan & Bennett, 2008; Marko *et al*., 2010; Lumibao *et al*., 2017; Hošek *et al*., 2024). Cutting‐edge spatial inference with ARGs has so far focused on humans and Arabidopsis (Wohns *et al*., 2022; Osmond & Coop, 2024; Grundler *et al*., 2025), and we exemplify how these techniques can provide detailed insights into phylogeographic questions in non‐model taxa. Finally, our analysis of *Macrocystis* highlighted how genetic relationships among morphs are inconsistent across different global regions, including within North America, such that a full taxonomic treatment using genomic samples from all morphs and geographic regions is warranted.

## Competing interests

None declared.

## Author contributions

JBB and GLO conceptualized the study. JBB and MMO performed the formal analysis. RAB, SCL, MMO and GLO acquired the funding. JBB conducted the investigation. KJK, SRP, RAB, KMG and SCL provided the resources. MMO provided the software. GLO supervised the study. JBB wrote the original draft of the manuscript. GLO reviewed and edited the manuscript with contributions from all authors.

## Disclaimer

The New Phytologist Foundation remains neutral with regard to jurisdictional claims in maps and in any institutional affiliations.

## Supporting information


**Fig. S1** Map of geographic features mentioned in the text.
**Fig. S2** Impact of sequencing depth on California *Macrocystis* PCA.
**Fig. S3** Relationship between sequencing depth and genetic diversity.
**Fig. S4** Genetic divergence and differentiation.
**Fig. S5** Isolation by distance.
**Fig. S6** Phylogenetic relationships and gene flow among global *Macrocystis*.
**Fig. S7** Occurrence records used in ecological niche models.
**Fig. S8** Habitat suitability in the current time period.
**Fig. S9** Habitat suitability during the Last Glacial Maximum – ensemble model.
**Fig. S10** Habitat suitability during the Last Glacial Maximum – CCSM3 model.
**Fig. S11** Randomization test in *Spacetrees*.
**Fig. S12** Leave‐one‐out analysis in *Spacetrees*.
**Methods S1** Methodological details.
**Table S1** Sampling information for newly sequenced data.
**Table S2** Sampling information for previously published sequencing data.
**Table S3** Ecological niche model predictor variable importance metrics.
**Table S4** Ecological niche model performance metrics.
**Table S5** Divergence time estimates between populations.
Please note: Wiley is not responsible for the content or functionality of any Supporting Information supplied by the authors. Any queries (other than missing material) should be directed to the *New Phytologist* Central Office.

## Data Availability

Raw DNA sequences are openly available in the NCBI SRA at https://www.ncbi.nlm.nih.gov/sra, BioProject Numbers PRJNA1256528 and PRJNA1140647. Custom scripts used to process data are openly available in Zenodo at https://zenodo.org, doi: 10.5281/zenodo.19986475.
